# Rapid Global Expansion of the Fungal Disease Chytridiomycosis into Declining and Healthy Amphibian Populations

**DOI:** 10.1371/journal.ppat.1000458

**Published:** 2009-05-29

**Authors:** Timothy Y. James, Anastasia P. Litvintseva, Rytas Vilgalys, Jess A. T. Morgan, John W. Taylor, Matthew C. Fisher, Lee Berger, Ché Weldon, Louis du Preez, Joyce E. Longcore

**Affiliations:** 1 Department of Biology, Duke University, Durham, North Carolina, United States of America; 2 Department of Ecology and Evolutionary Biology, University of Michigan, Ann Arbor, Michigan, United States of America; 3 Department of Molecular Genetics and Microbiology, Duke University Medical Center, Durham, North Carolina, United States of America; 4 Department of Primary Industries & Fisheries, Animal Research Institute, Yeerongpilly, Queensland, Australia; 5 Department of Plant and Microbial Biology, University of California at Berkeley, Berkeley, California, United States of America; 6 Imperial College Faculty of Medicine, Department of Infectious Disease Epidemiology, St. Mary's Campus, London, United Kingdom; 7 School of Public Health, Tropical Medicine and Rehabilitation Sciences, James Cook University, Townsville, Queensland, Australia; 8 School of Environmental Sciences and Development, North-West University, Potchefstroom, South Africa; 9 School of Biology & Ecology, University of Maine, Orono, Maine, United States of America; University of Birmingham, United Kingdom

## Abstract

The fungal disease chytridiomycosis, caused by *Batrachochytrium dendrobatidis*, is enigmatic because it occurs globally in both declining and apparently healthy (non-declining) amphibian populations. This distribution has fueled debate concerning whether, in sites where it has recently been found, the pathogen was introduced or is endemic. In this study, we addressed the molecular population genetics of a global collection of fungal strains from both declining and healthy amphibian populations using DNA sequence variation from 17 nuclear loci and a large fragment from the mitochondrial genome. We found a low rate of DNA polymorphism, with only two sequence alleles detected at each locus, but a high diversity of diploid genotypes. Half of the loci displayed an excess of heterozygous genotypes, consistent with a primarily clonal mode of reproduction. Despite the absence of obvious sex, genotypic diversity was high (44 unique genotypes out of 59 strains). We provide evidence that the observed genotypic variation can be generated by loss of heterozygosity through mitotic recombination. One strain isolated from a bullfrog possessed as much allelic diversity as the entire global sample, suggesting the current epidemic can be traced back to the outbreak of a single clonal lineage. These data are consistent with the current chytridiomycosis epidemic resulting from a novel pathogen undergoing a rapid and recent range expansion. The widespread occurrence of the same lineage in both healthy and declining populations suggests that the outcome of the disease is contingent on environmental factors and host resistance.

## Author Summary

Amphibian species are facing a current global extinction crisis of unprecedented magnitude. The major factors causing their decline are the emerging disease chytridiomycosis and habitat destruction. Chytridiomycosis is caused by the aquatic fungus *Batrachochytrium dendrobatidis* and has been linked to species extinctions and population declines in montane regions including Australia, Panama, North America, and Spain. Currently, it is debated whether the recent emergence of the pathogen is largely the result of environmental factors triggering an outbreak of an endemic pathogen or if the epidemic has been caused by widespread introduction of the pathogen into naïve host populations (“pathogen pollution”). We studied the population genetics of chytridiomycosis using DNA sequences from a global panel of strains. These data showed evidence of a strong genetic bottleneck in the history of the pathogen, and the epidemic appears traceable to the widespread dispersal of a single genotype. Populations were not structured by host-origin, and the same lineage was detected in populations of both resistant and highly sensitive species. The data suggest that the chytridiomycosis epidemic is caused by the emergence of a novel pathogen but that disease outcome is contingent on host resistance and environmental factors.

## Introduction

Globally, amphibian species are threatened by the emergence of a novel pathogen, the chytrid fungus *Batrachochytrium dendrobatidis* (*Bd*) [1],[2]. The pathogen proliferates in epidermal cells of amphibians leading to hyperkeratosis, electrolyte loss [1],[3], and ultimately death in susceptible species. The precise cause of osmotic imbalance and death is uncertain, but is hypothesized to be caused by physical skin damage or unidentified toxins produced by the pathogen [3],[4]. Some species, such as the North American bullfrog, show no evidence of morbidity and are likely to have acted as vectors by facilitating the widespread dispersal of the pathogen and by providing a reservoir of infection [5],[6],[7]. Amphibian declines caused by chytridiomycosis have not affected all areas of the planet homogeneously but are well documented in montane regions of Australia, western North America, Panama, and Spain [1],[8],[9],[10],[11]. In other regions such as eastern North America and South Africa, chytridiomycosis occurs but has not been linked to declines [12],[13],[14].

The disease chytridiomycosis was only recently discovered [1],[15],[16], and debate centers on whether *Bd* is an endemic pathogen whose emergence is due to recent changes in the environment versus the alternative that *Bd* is a novel pathogen introduced into naïve host populations, or a combination of the two [9],[17],[18],[19],[20]. Analyses of the pattern of declines in Australia [21],[22], Panama [23], and California [24] provided convincing evidence of recent spread of the pathogen, consistent with the idea that the pathogen is novel in these areas. Histological examinations of museum specimens also support a recent origin of the disease, with the oldest known infections dating to 1938 on *Xenopus laevis* from Africa [25]. In contrast, the endemic pathogen hypothesis has been invoked to explain the correlation between harlequin frog (*Atelopus* spp.) extinctions in Central America and global climate change measured as sea surface temperature [18].

Despite growing evidence that the disease is increasing in geographic range, no clear evidence points to a source population of chytridiomycosis. Two hypotheses explain how *Bd* may have been spread across the globe but remain to be tested. One postulates that the disease evolved in Africa, being thereafter transported intercontinentally through dissemination of *Xenopus* for aquaria, scientific research, and pregnancy assays [25]. A second proposes that the disease spread through purposeful and unintentional range expansions of the North American Bullfrog *Rana catesbeiana* [6]. *R. catesbeiana* is native to eastern North America but has been widely introduced to Asia, Europe, western North America and South America [26]; its current distribution cannot fully explain the arrival of *Bd* in regions such as Australia and South Africa.

The inability to disentangle the endemic from the novel pathogen hypotheses stems from the fact that little is known about the global population genetic structure of *Bd*. Previous studies of *Bd* have suggested low genetic variation in the pathogen and widespread dispersal of closely related pathogen genotypes [10],[27]. However, the only broadly sampled global study to date included only three polymorphic loci, making it difficult to infer statistically significant patterns in the data. Several questions must be addressed to resolve the ongoing debate: is there evidence for population structure within *Bd*, how is genetic diversity generated, is there evidence for a source population, and do *Bd* isolates from healthy amphibian populations (defined in this study as species or geographic regions having no evidence of population declines) have the same genetic structure as those from declining populations? The central prediction of the endemic hypothesis is that geographic population structure will be detected because populations are at equilibrium. In contrast, the novel pathogen hypothesis predicts geographically widespread genotypes and the occurrence of a genetic bottleneck resulting from the rapid range expansion of the disease. A key assumption is that source populations can be identified because they harbor greater genetic diversity [28],[29],[30].

In this study we address these questions of genetic diversity in *Bd* with 59 strains isolated from five continents and 31 host species (Table 1) and 17 polymorphic nuclear loci. We also sequenced a non-coding region (>11 kbp) of the mitochondrial genome for a subset of 16 strains. These data were used to test the predictions of the endemic versus the novel pathogen hypotheses. Genetic diversity and heterozygosity of the diploid pathogen were compared between various subpopulations to test for geographic structure and to identify if any were a source of the current epidemic. Consistent with the novel pathogen hypothesis, we observed an extremely low allelic diversity in the pathogen, suggesting a severe bottleneck during the current epidemic. The data also demonstrate that the pathogen reproduces primarily asexually, but that genotypic diversity is high. Using information on the genomic location of marker loci we show how this paradox can be reconciled by invoking loss of heterozygosity as a product of mitotic recombination.

**Table 1:** Origin of strains of *B. dendrobatidis* used in this study.

Strain	Geographic Origin	Host	Health
Al99LB = Alstonville-Lcaerulea-99-LB-1	Alstonville, New South Wales, Australia	*Litoria caerulea*	clinical
CW-026	Namaqualand, South Africa	*Amietia fuscigula*	aclinical
CW-027	Namaqualand, South Africa	*Amietia fuscigula*	aclinical
CW-029	Namaqualand, South Africa	*Xenopus laevis*	aclinical
CW-034	Namaqualand, South Africa	*Xenopus laevis*	aclinical
CW-036	Port Elizabeth, South Africa	*Amietia fuscigula*	infected tadpole^1^
EUR042	Pyrenees, Spain	*Alytes obstetricans*	dead
EUR043	Pyrenees, Spain	*Alytes obstetricans*	dead
EURC2A	Sierra de Guadarrama, Spain	*Alytes obstetricans*	dead
JAM011	Mono Pass, California, USA	*Rana muscosa*	infected tadpole
JAM018	Mono Pass, California, USA	*Rana muscosa*	infected tadpole
JAM033	Summit Meadow, California, USA	*Rana muscosa*	infected tadpole
JAM050	Hitchcock Lakes, California, USA	*Rana muscosa*	infected tadpole
JAM083	Little Indian Valley, California, USA	*Rana muscosa*	infected tadpole
JAM084	Little Indian Valley, California, USA	*Rana muscosa*	infected tadpole
JAM102	Woods Lake, California, USA	*Rana muscosa*	infected tadpole
JEL197	National Zoological Park, DC, USA	*Dendrobates azureus*	dead
JEL198	National Zoological Park, DC, USA	*Dendrobates auratus*	dead
JEL203	Bronx Zoo, New York, USA	*Dyscophus guineti*	dead
JEL213	Mono Co., California, USA	*Rana muscosa*	aclinical
JEL225	Africa (from captive population in Wisconsin, USA)	*Silurana tropicalis*	aclinical
JEL226	Yavapai Co., Arizona, USA	*Rana yavapaiensis*	dead
JEL229	Montrose Canyon, Arizona, USA	*Hyla arenicolor*	dead
JEL230	Montrose Canyon, Arizona, USA	*Rana yavapaiensis*	dead
JEL231	Mesquite Wash, Arizona, USA	*Rana yavapaiensis*	dead
JEL239	Ghana (imported)	*Silurana tropicalis*	aclinical
JEL253	Melbourne, Victoria, Australia (captive)	*Limnodynastes dumerilii*	clinical
JEL254	Orono, Maine, USA	*Rana pipiens*	roadkill
JEL258	Orono, Maine, USA	*Rana sylvatica*	roadkill
JEL262	Quebec, Canada	*Rana catesbeiana*	aclinical
JEL270	Point Reyes, California, USA	*Rana catesbeiana*	aclinical
JEL271	Point Reyes, California, USA	*Rana catesbeiana*	aclinical
JEL273	Clear Creek Co., Colorado, USA	*Bufo boreas*	clinical
JEL274	Clear Creek Co., Colorado, USA	*Bufo boreas*	clinical
JEL275	Clear Creek Co., Colorado, USA	*Bufo boreas*	aclinical
JEL277	Arizona, USA	*Ambystoma tigrinum*	aclinical
JEL282	Toledo Zoo, Ohio, USA	*Bufo americana*	clinical
JEL284	Wisconsin, USA (captive)	*Rana pipiens*	aclinical
JEL289	Milford, Maine, USA	*Rana pipiens*	roadkill
JEL404	Crocker Pond, Oxford County, Maine	*Rana catesbeiana*	infected tadpole
JEL408	El Cope, Panama	*Colostethus inguinalis*	clinical
JEL409	Silenciosa, Panama	*Eleutherodactylus talamancae*	dead or clinical
JEL415	between Loop and Silenciosa, Panama	*Eleutherodactylus podi-noblei*	dead or clinical
JEL423	Guabal, Panama	*Phyllomedusa lemur*	dead or clinical
JEL424	Loop trail, Panama	*Cochranella euknemos*	dead or clinical
JEL425	El Cope, Panama	*Bufo haematiticus*	dead or clinical
JEL427	Puerto Rico	*Eleutherodactylus coqui*	aclinical
JEL429	Venezuela	*Rana catesbeiana*	aclinical
JP005	Berkeley, California, USA	*Xenopus laevis*	aclinical
LJR089	Laurel Creek, California, USA	*Rana muscosa*	infected tadpole
LJR299	Point Reyes, California, USA	*Rana aurora draytonii*	dead or clinical
Me00LB = Melbourne-Llesueuri-00-LB-1	Melbourne, Victoria, Australia (captive)	*Litoria lesueuri*	clinical
MM06LB = MtMisery-Lrheocola-06-LB-1	Mt. Misery, Queensland, Australia	*Litoria rheocola*	dead
PM-01	Panama	*Eleuthodactylus caryophyllaceum*	dead
PM-05	Panama	*Smilisca phaeota*	dead
PM-07	Panama	*Smilisca phaeota*	dead
Ro99LB = Rockhampton-Lcaerulea-99-LB-1	Rockhampton, Queensland, Australia	*Litoria caerulea*	clinical
To05LB = Townsville-Lcaerulea-05-LB-1	James Cook University, Queensland, Australia (captive)	*Litoria caerulea*	clinical
Tu98LB = Tully-Ndayi-98-LB-1	Tully, Queensland, Australia	*Nyctimystes dayi*	clinical

Health refers to the status of the host animal at the time of sampling.

1 Strain isolated from a tadpole. Infected tadpoles do not usually die until metamorphosis.

## Results/Discussion

Multilocus sequence typing (MLST) of *Bd* strains confirmed that the fungus is diploid because all strains were heterozygous at multiple loci. Despite the geographic breadth of sampling, only two alleles (sequence variants) were detected at each of the 17 loci. The limited allelic variation is not restricted to the nuclear genome. Zero nucleotide polymorphisms were found in the >11 kbp portion of the mitochondrial genome surveyed in the subsample of global strains, though one large deletion of 3,904 bp occurred in 4 of the 16 strains. This low genetic variation is consistent with a previous study describing minimal global genetic variation using fewer genetic markers and sparser sampling [27]. The previous study found that 7 of 10 loci were monomorphic; in this study only polymorphic loci were included.

### Host and geographic patterns of population substructure

**Figure 1:**
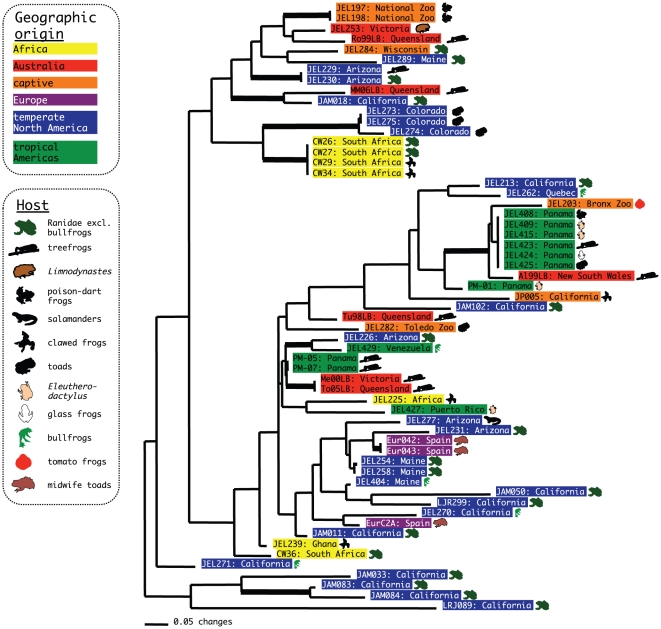
Dendrogram depicting relationships among *Bd* strains. The tree was computed using neighbor-joining in PAUP v4.0b10 [79] with “hetequal” coding, and the thickened branches indicate bootstrap values of 50% or greater.

We analyzed the genetic similarity of isolates with MLST data to determine the association between genotype and geographic origin (Figure 1). Strains from the same geographic region showed some tendency to cluster together, including several strains of identical multilocus genotype (Figure 1). Larger clusters of strains with identical genotypes are one Panamanian group of six strains and a group of four strains from South Africa. An association between strains from Spain and the United States was also detected. However, broad geographic structure is limited; for example, strains from Australia and Africa are found in several unrelated portions of the dendrogram. Few branches in the dendrogram are supported by bootstrap resampling, presumably due to the limited resolving power of two alleles per sequenced locus. Because of the likelihood of homoplasy among multilocus genotypes due to recombination, a phenetic method (neighbor joining) for tree reconstruction was used rather than a cladistic method. Network approaches can account for this homoplasy by introducing loops where recombination could have occurred in the history of the genealogy. A network of the multilocus genotypes derived using statistical parsimony was constructed and revealed a very similar pattern of genotypic relatedness as the neighbor-joining tree (Figure S1), including the close association of Spanish and the United States strains.

The absence of an overarching geographic structure to the dendrogram could result from low resolving power of the markers, recombination among loci, or gene flow between regions. Although recombination has occurred in the history of the *Bd* epidemic [10], the marker loci do possess a phylogenetic signal as estimated by the permutation tail probability test. The shortest trees estimated by parsimony for the non-permuted dataset were 118 (“hetequal” coding), whereas the mean tree length of 100 permuted datasets was 217.1 (P<0.01). This test remained highly significant after removing repeated genotypes (clone-correction). These data suggest that recombination among loci has not obliterated the phylogenetic signal among genotypes, and that the absence of a broad geographic pattern (Figure 1) lends support to the novel pathogen hypothesis during which long distance dispersal has occurred.

To test whether significant geographic structuring exists between regions, strains were grouped by geographic origin and were tested for significant differences in allele frequency. These tests identified significant differences (P<0.05) between strains from tropical America and all other populations. Temperate American strains were also statistically differentiated from African strains. However, following clone-correction of the dataset by eliminating strains with repeated genotypes, only the difference between temperate American versus tropical American strains remained significant (P<0.05). The power to detect additional genetic structure within the global sample is likely to be limited by low numbers of isolates from poorly sampled regions such as Europe. Nonetheless, some alleles (BdC24, 9893X2, and R6046) were unique to temperate America and Europe and were never detected in African, Australian, or tropical American strains. However, until additional samples are obtained from outside North America, the absence of these alleles may be due to sampling error. Altogether, these data indicate substructure within the global *Bd* population, implying that contemporary intercontinental gene flow is not prevalent. Instead, the MLST population structure of our isolates is consistent with a single, or a few, introductions between regions followed by population differentiation.

Visual inspection of the dendrogram (Figure 1) shows little effect of host species on pathogen relatedness, suggesting no host specificity of pathogen genotypes. The six strains from Panama of identical genotype were each isolated from a different host species. The stronger geographic than host pattern confirms results from field surveys showing that *Bd* is a pathogen that undergoes rapid local proliferation with low host specificity [2],[17],[31]. Neither isolates from *Xenopus* nor from *R. catesbeiana* formed a cluster and instead were scattered throughout the dendrogram. Although host species is not a good indicator of genetic relatedness or virulence, recent evidence suggests that strains may differ in their virulence [32],[33],[34]. In laboratory trials, strain Melbourne-Llesueuri-00-LB-1 (Me00LB) caused more rapid mortality in *Litoria caerulea* than strain Rockhampton-Lcaerulea-99-LB-1 (Ro99LB) [32]. These strains are in separate parts of the dendrogram and differ in genotype at 10 loci. Furthermore, a recent study demonstrated that virulence towards *Bufo bufo* and other phenotypic traits of the pathogen, such as sporangia size, were correlated with genetic relatedness between strains as assessed using the same MLST markers employed in this study [34]. Future studies that address the relative virulence of a large number of *Bd* strains on a single common model host species (e.g., *Bufo bufo*) will be required to determine the evolution of virulence in *Bd* and to reconcile differences in virulence observed in various host by strain inoculation studies [34],[35]. These data may help explain the observation of widespread and closely related genotypes found in regions such as Panama and Australia (Figure 1).

### Host and geographic patterns of genetic diversity

If *Bd* has been recently dispersed from a geographic-restricted or host-restricted source population, that source population is expected to harbor greater genetic diversity than populations where the pathogen is newly established. Furthermore, if *Bd* has been stochastically spread from region to region via long distance dispersal, then each introduction is likely to have caused a concurrent bottleneck in genetic diversity. Genetic diversity can be expressed as “allelic diversity” (number of alleles per locus), “genotypic diversity” (number of unique genotypes in a sample), or as “gene diversity” (expected heterozygosity under Hardy Weinberg equilibrium [HWE] or *H_E_*). The difference between genotypic diversity and gene diversity relates to the manner of reproduction; for example, clonal reproduction leads to a proliferation of strains of the same genotype. In the present study, we found a striking lack of allelic diversity. “Global” allele diversity was limited to two alleles per locus, and was even lower in Africa, tropical America, and Australia where the second alleles at BdC24, 9893X2, and R6046 have never been detected (Table 2). In contrast, genotypic and gene diversity were high; the 59 strains were of 44 multilocus genotypes, with a global *H_E_* of 0.468 (Table 2). Geographic regions differed little in the number of genotypes recovered per strain sequenced but did differ in *H_E_*, with strains from Africa, Australia, and tropical America having lower gene diversity (Table 2). The lower heterozygosity (*H_E_* and *H_O_*) in tropical American (primarily Panamanian) and Australian populations is consistent with a bottleneck and epidemic spread in these regions that have undergone some of the most severe population declines and pathogen expansions [1],[17]. An alternative explanation is that the geographic breadth of these populations is significantly smaller than that sampled in temperate North America.

**Table 2:** Heterozygosity of *B. dendrobatidis* at 17 sequenced loci.

Comparison	Population	Expected Het. (*H_E_*)	Observed Het. (*H_O_*)	Allele richness	# strains	# genotypes
*Geography*	Global	0.468	0.552	1.98	59	44
	Temperate Americas	0.488	0.575	2.00	25	22
	Tropical Americas	0.348	0.532	1.80	11	5
	Australia	0.419	0.504	1.82	7	6
	Africa	0.360	0.529	1.80	7	4
	captive	0.467	0.431	1.98	6	5
	Europe	0.558	0.833	–	3	2
*Host*	bullfrog strains	0.525	0.729	2.00	5	5
	clawed frog strains	0.427	0.518	1.82	5	4
	other hosts	0.468	0.537	1.96	49	36
*Health*	morbid/dead hosts	0.453	0.543	1.99	30	20
	aclinical/subclinical hosts	0.461	0.548	2.00	16	12

Allele richness is the number of alleles per locus calculated using rarefaction with FSTAT v2.9.3.2 [83]. Samples were rarefied to 5 diploid individuals for geographic comparisons, 4 diploid individuals for host comparisons, and 15 diploid individuals for health comparisons.

Measures of allelic and genotypic diversity of *Bd* strains were not different between healthy (aclinical or subclinical) and sick or dying hosts presenting clinical symptoms of chytridiomycosis (Table 2). Over all host species, *H_E_* of strains isolated from aclinical hosts was similar to that of strains from clinical or dead hosts (0.461 vs. 0.453); these differences were not significantly different when tested by permuting samples between groups (P = 0.502). It should be noted, however, that the incubation period of chytridiomycosis may be as long as two months [36], meaning that classification of animals into sick and aclinical groups can be confounded. Nonetheless, these data provide evidence that there are not highly virulent strains that cause mortality that are a separate genetic pool from strains isolated from highly resistant hosts (e.g., *Xenopus*).

Comparisons of diversity among host species revealed that the *H_E_* and *H_O_* of strains from clawed frogs (*Xenopus*) was lower than the global mean, whereas that of bullfrog strains was greater than the global mean. Despite the limited sampling of isolates from both of these hosts, permutation tests suggested that the increased diversity (*H_E_*) among bullfrog strains relative to other hosts was significantly greater than random expectations (P = 0.041), whereas *Xenopus* diversity was not significantly lower (P = 0.073). *Xenopus* spp. and the related genus *Silurana* are found throughout sub-Saharan Africa and appear to have originated in central or eastern equatorial Africa [37]. However, in the present study few African strains and only ones from South Africa and Ghana were included, implying that additional samples encompassing more geographic and species diversity of *Xenopus* will be required to fully test the “out of Africa” hypothesis for the origin of the *Bd* pandemic. The absence of increased genetic diversity in African clawed frog samples suggests that *X. laevis* and *S. tropicalis* may not be a source population, but these data do not effect the hypothesis that clawed frogs were a vector of the disease from Africa through amphibian exportation.

### Evidence for both clonality and recombination

Asexual reproduction is the only form of *Bd* reproduction documented in the lab, and there is no morphological evidence of sexual reproduction in nature. Examination of molecular genotype data has produced multiple hypotheses on the role of recombination in shaping the population genetics of *Bd*. Recovery of multiple strains of identical genotype and an excess of heterozygosity at some loci in a global sample of strains suggested outcrossing to be rare or absent [27], whereas genotype distributions in the Sierra Nevada of California were consistent with both clonality within a site, e.g., lake, and recombination occurring in the history of divergence between lakes [10]. Previous results of fixed heterozygosity for some *Bd* loci were also coupled with the observation that other loci show HWE proportions of genotypes, a contradiction that was reconciled by invoking a recombination process that is not homogenous throughout the genome [27]. By now including additional MLST loci from a broader sampling of strains and by mapping the loci onto their genomic location, we have determined that genotypic diversity of many strains can be explained as the result of loss of heterozygosity (LOH) that occurs during asexual reproduction, a mechanism that does not involve outcrossing.

Although none of the marker loci showed completely fixed heterozygosity, 7 of the 17 markers had heterozygote excess (negative inbreeding coefficients or *F_IS_*) significantly greater than HWE expectations (Table 3; P<0.01). Three of the loci with positive inbreeding coefficients (*F_IS_*) showed significant heterozygote deficiency (P<0.05). After applying a clone-correction (elimination of repeated genotypes) all of the loci displaying heterozygote excess were still significantly different from HWE, whereas only one of the loci showing heterozygote deficit (9893X2) was significant. This test for heterozygote excess is highly conservative because the test was computed for the entire global dataset. When data from multiple subpopulations are analyzed together, the Wahlund effect [38] increases homozygosity, even for randomly mating populations. The Wahlund effect probably explains the significant heterozygote deficit of 9893X2, where the second allele has been detected only in North America and Europe.

**Table 3:** Observed heterozygosity is related to position of the locus in the genome.

Locus	Supercontig	Position (bp)	*H_O_*	*F_IS_*	Test of HWE	Test of HWE (clone corrected)
6873X2	1.1	314,229	0.328	0.336	0.0149	0.1194
8392X2	1.1	359,794	0.356	0.283	0.0361	0.2317
8009X2	1.1	636,628	0.390	0.220	0.1159	0.5491
6677X2	1.1	726,612	0.431	0.144	0.3014	1.0000
b7-10c	1.1	824,826	0.424	0.160	0.2963	1.0000
BdC5	1.1	1,449,098	0.441	0.115	0.4333	0.7553
8329X2	1.1	1,603,575	0.559	−0.111	0.4406	0.3801
BdC24	1.1	2,750,438	0.390	−0.159	0.4297	0.2392
9893X2	1.1	4,310,256	0.123	0.564	0.0003	0.0015
APRT13	1.5	766,452	0.978	−0.957	0.0000	0.0000
R6046	1.5	1,217,203	0.254	0.247	0.1070	0.0959
8702X2	1.9	139,500	0.729	−0.478	0.0004	0.0002
6164Y2	1.10	272,218	0.814	−0.622	0.0000	0.0003
mb-b13	1.10	409,375	0.847	−0.695	0.0000	0.0000
BdC18.2	1.11	461,391	0.746	−0.521	0.0001	0.0067
BdC18.1	1.11	461,770	0.746	−0.489	0.0002	0.0177
CTSYN1	1.15	117,731	0.831	−0.664	0.0000	0.0000

*F_IS_* is the inbreeding coefficient that ranges from −1 (fixed heterozygosity) to +1 (complete heterozygote deficit). Position refers to the location within a supercontig in Assembly 1 (September 7, 2006) of the first variable position of the locus. Supercontigs are ordered in descending size from 1.1 (2.38 Mbp) to 1.69 (0.56 Mbp). The test of HWE is the probability that data are drawn from a population in Hardy Weinberg equilibrium, calculated using an exact test [84] as implemented in Genepop v. 3.4 [81]. The test of HWE was conducted for both uncorrected and clone-corrected data sets. Significant values of the test are P<0.05.

The recent sequencing of the genomes of two strains of *Bd* by the Broad Institute and the Joint Genome Institute now allows these marker loci to be placed into a genomic context. The genome of JEL423 has been assembled into 69 supercontigs: 10 are greater than 1 Mbp, and the largest is 4.44 Mbp (http://www.broad.mit.edu/annotation/genome/batrachochytrium_dendrobatidis). Chromosome sizes in *Bd* were shown by pulsed-field gel electrophoresis to range from ∼0.7–6.0 Mbp [27] and thus the largest supercontig probably represents a majority of the largest chromosome. Mapping our MLST markers onto their genomic location revealed that all markers on the smaller supercontigs except R6046 had an excess of heterozygotes (>70% observed heterozygosity; significantly different from HWE expectations [P<0.05]), whereas 7 of 9 marker loci on the largest supercontig had higher numbers of homozygous genotypes (positive *F_IS_*), and none of the loci displayed significant heterozygote excess (Table 3).

**Figure 2:**
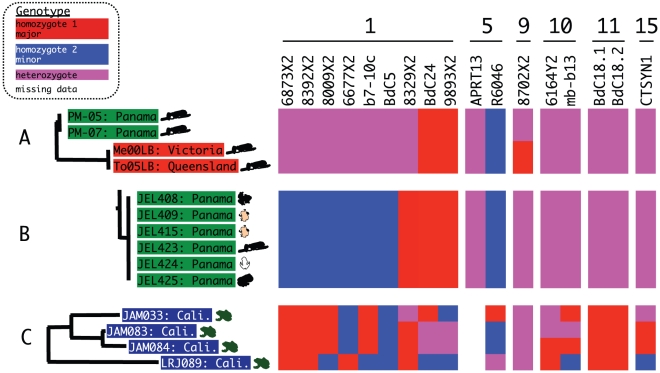
Loss of heterozygosity (LOH) among closely related strains. Genotypes for each locus are purple for heterozygous genotypes, red for the higher frequency homozygous genotype, blue for the minority homozygous genotype, and white for missing data. Locus names are shown above the genotypes and are ordered into linkage groups of descending supercontig size based on comparison to genome supercontigs (number shown above the locus names) from the assembly of strain JEL423 (http://www.broad.mit.edu/annotation/genome/batrachochytrium_dendrobatidis). For precise genomic locations, see Table 3. (A) Pattern of LOH showing closely related strains from Panama (green highlighting) and Australia (red highlighting) differing in genotype at a single locus (8702X2). (B) Prevalent genotype from Panama differing from Panamanian genotype shown in (A) by LOH of all markers on supercontig 1.1. (C) Genotypes of strains from the Sierra Nevada showing both LOH (compare JAM083 and JAM084) and highly recombined genotypes (JAM033 and LRJ089).

These data suggest that heterozygosity is not uniform across the genome but is reduced on the largest chromosome. Such a pattern is not consistent with frequent sexual reproduction and independent assortment of chromosomes [39] but could be explained by chromosome-specific variation in mitotic recombination or aneuploidy causing loss of heterozygosity (LOH) within diploid individuals. Selective loss of specific chromosomes, such as chromosome 5 in *Candida albicans* strains adapting to sorbose containing media [40], as well as a general relationship between chromosome size and number of sister chromatid exchanges [41], should both create heterogeneity in rates of LOH among chromosomes. At the population level, chromosome-specific levels of heterozygosity have also been observed in alloploid hybrids of the fungal pathogen *Cryptococcus neoformans* between varieties *neoformans* and *grubii*, “serotype AD hybrids.” These hybrids are unable to complete normal meiotic reduction, and these naturally occurring diploid or aneuploid strains appear to have retained heterozygosity on specific chromosomes while other chromosomes show higher degrees of homozygosity [42]. Although mitotic recombination can only eliminate variation by reducing heterozygosity at certain loci, it has great power to generate genotypic diversity, and this genotypic diversity could facilitate adaptation by exposing beneficial recessive alleles and by increasing the rate of fixation of beneficial mutations [43],[44],[45]. LOH in diploid pathogens has also been implicated in attenuation of virulence and acquisition of drug resistance [46],[47],[48]. Specific evidence for a role of LOH in generating genotypic differences among *Bd* strains is found in several strain clusters (Figure 2A) that differ only by heterozygosity at a single locus or linked loci (e.g., the two related genotypes PM-05+PM-07 and Me00LB+To05LB differ only in heterozygosity of the marker 8702X2 on supercontig 1.9; Figure 2A). LOH of all of the markers on supercontig 1.1 was found when the six strains of identical multilocus genotype from Panama (JEL408, JEL409, JEL415, JEL423, JEL424, JEL425; Figure 2B) were compared with the two strains from Panama collected a few years earlier (PM-05, PM-07, Figure 2A).

We tested the fit of a completely non-outcrossing model for the *Bd* genotype data by inferring ancestral recombination events that occurred during the spread of the epidemic. The genealogy of the strains was assumed to be the same as the dendrogram shown in Figure 1, and the ancestral genotypic states for each locus were inferred using parsimony [49]. Under a completely asexual mode of reproduction (with recombination occurring solely by LOH), a single genealogy should be compatible with all of the loci because genomes are inherited without admixture [50]. Alternatively, where ancestral state transitions occur from one homozygous genotype to the other homozygous genotype or from a homozygous genotype to a heterozygous genotype, genotypic changes must have occurred as the result of outcrossing or mutation. The number of outcrossing/mutation events needed to reconcile the neighbor-joining tree shown in Figure 1 range from 0–6 for each locus. Over half (9/17) of the loci were completely compatible with a LOH-only mode of recombination (zero inferred outcrossing/mutation events). However, results for the remaining 8 loci suggest that sex may have occurred in the history of the epidemic. The possibility of sex in *Bd* has important epidemiological consequences as sex in the chytrid fungi always leads to the production of a resistant sporangium stage that could facilitate long distance dispersal and survival during hostile seasons. In contrast, the inferred outcrossing events could merely be due to the imprecise estimation of the genealogy or the failure to accommodate linkage into our LOH model. Distinguishing among true outcrossing and a mitotic-only recombination system will require typing a much more extended set of marker loci. Currently, the nearly-fixed heterozygosity of multiple loci in the global population favors the hypothesis that meiosis has not occurred during the current pandemic, perhaps due to genome-wide heterosis.

The population structure and basic genetics of *Bd* shares a number of similarities with the fungus *Candida albicans*, a species that is normally a commensal of the gastrointestinal and genitourinary tract biota but also an important opportunistic pathogen. Both are diploid and the population genetic structures of both show a high level of heterozygosity and a primarily asexual mode of reproduction [45],[51],[52],[53],[54]. Yet, populations of both species harbor extensive genotypic diversity [45],[55], and in *C. albicans* it has been suggested that a large proportion of genotypic diversity results from LOH during mitotic division [45],[56]. In a recent population genetic analysis of *C. albicans* strains, a negative correlation was observed between distance of the locus to the centromere and number of sites per locus displaying heterozygote excess [45]. Heterozygosity is expected to decline with increasing distance to the centromere because exchange between sister chromatids during mitosis causes LOH of all loci distal to the breakpoint [43],[57]. Such a pattern was observed in the genome sequence of chromosome 7 of *C. albicans* strain SC5314 [58], and commensal *C. albicans* isolates also undergo a process of LOH by sister chromatid exchange in which all markers distal to the breakpoint became homozygous [59]. Centromeres have not been located for *Bd*, but the data indicate that, at least for the largest chromosome (supercontig1.1), *H_O_* is highest in the middle (Table 3), possibly where the centromere would be located.

A model explaining all genotypic diversity for *Bd* by LOH may not be parsimonious for all portions of the globe. Sierra Nevada populations are at the edge of a current epidemic, yet *Bd* strains from this region encompass a wide range of *H_O_* [10]. California strains (LRJ089, JAM033, JAM083, JAM084; Figure 2C) show evidence of genotypic divergence by LOH (compare JAM083 to JAM084) as well as highly recombined genotypes that would require short gene conversion tracts or double crossovers (JAM033 and LRJ089; Figure 2C). A comparison of commensal strains of *C. albicans* obtained from the same patient or patient family has revealed three mechanisms for LOH between strain pairs: sister chromatid exchange, chromosomal loss and reduplication, and short tracks of gene conversion [59]. Interestingly, the mechanism depended on both the strain pair and the chromosome investigated, suggesting LOH mechanisms may vary depending on the physical structure and chromosomal gene/locus constituency. In *C. albicans* mating can occur between diploid strains homozygous for alternate MAT alleles followed by random chromosome loss including homologous recombination to revert back to a diploid genome [60],[61]. In tetraploid products of the same fungus, Forche et al. [61] found that homologous chromosomes commonly experienced multiple recombination/gene conversion events during the completion of the parasexual cycle. The possibility for a parasexual cycle involving diploid-diploid cell fusions in *Bd* is an interesting possibility, but has been unexplored. One possibility that may explain differences in mode of recombination among populations of *Bd* is that some populations, such as those from Panama and Australia, lack sexual reproduction, whereas other populations, such as California, may be capable of outcrossing. These differences in mating system among populations could be from the loss of complementary mating types and outcrossing ability through genetic bottlenecks in regions where *Bd* has been recently introduced; unfortunately knowledge of mating types in Chytridiomycota is essentially nonexistent. Alternatively, the differences in pattern may be due to different routes of LOH in these areas, e.g., sister chromatid exchange in Panama and short gene conversion tracts or outcrossing between very closely related strains in California.

### The ancestral genotype of the pandemic

These findings of an excess of heterozygosity throughout the genome but only two alleles per locus suggests that a single diploid lineage of *Bd* has recently expanded throughout global amphibian populations; this pattern is consistent with the novel pathogen hypothesis. The question remains as to why the fungus has so rapidly spread through apparently healthy populations. Did the pathogen emerge because of a chance dispersal event from a still uncertain source population that allowed a single lineage to exploit novel habitats? Under this hypothesis *Bd* would be ancestrally diploid and asexual and the pandemic is merely due to dispersal into a novel niche. An alternative scenario is that emergence occurred after an initial diploidization/hybridization event followed by loss of sex and clonal proliferation. In this scenario the ancestral *Bd* population was haploid and may or may not have been sexual. This hypothesized scenario is realized in *Cryptococcus neoformans* serotype AD hybrids which display hybrid vigor but may have attenuated virulence [62],[63]. The *C. neoformans* hybrids are nonetheless commonly found in the clinic and environment but are hybrid-sterile and unable to complete meiosis [64],[65]. The low amount of variation observed across the *Bd* genome does not support an allopolyploid hybridization event [27]. However, if the ancestral population had zygotic meiosis and a normally haploid life cycle like the majority of fungi, a diploidization event could be related to the origin of increased infectivity. Diploidization could be the result of an autopolyploidy due to spontaneous genome doubling. A final possibility is that *Bd* was ancestrally diploid and capable of both sexual and mitotic reproduction (as in most fungi) and that the current clonal epidemic is due to very strong selection on a single genotype. The fungus may be capable of outcrossing but this may be very rare and largely restricted to self-fusions or prevented from LOH by genome-wide heterosis. The absence of mating type control over self fusion of clonal gametes in Chytridiomycota and Blastocladiomycota has been discussed [66]. Currently we are exploring the potential for sex by investigating the polymorphism of genes that encode high mobility group-domain proteins that may be part of putative mating-type loci, as observed in another basal fungal lineage, Mucoromycotina [67]. Distinguishing among these competing hypotheses on the ancestral genome of the pandemic would be greatly facilitated by determining the ancestral ploidy of *Bd* through investigations of related chytrid species.

All of these theories on the origin of the ancestral genome of a pathogenic *Bd* predict that samples taken earlier in space or time during the spread of the clone should have the highest heterozygosity (*H_O_*). By such measures, North America, Europe, and bullfrog populations are potentially ancestral or sites of earlier introductions, with greater heterozygosity than other populations (Table 2). Our sampling regime was biased towards North America, the native range of the bullfrog, and the effects of this bias are that the population genetic parameters within non-North American populations are difficult to estimate and large pockets of genetic diversity may have been overlooked. Nonetheless, one strain (JEL404) isolated from a bullfrog tadpole from Maine, U.S.A., was heterozygous at all 17 of the loci investigated in this study. Thus, this single strain possesses all of the allelic diversity in the entire global sample, and each strain in the data set could, in theory, be derived from JEL404 by LOH. Two of the loci in this study were microsatellite markers, which are normally among the most polymorphic regions of the genome; this makes the finding of only two alleles per locus surprising given the current large population size predicted by the widespread distribution of the disease. If the global sample represents the expansion of a single widespread diploid lineage, this presents a conundrum because the same pandemic has spread through both declining and apparently healthy host populations.

We hypothesize that a mutation or genomic rearrangement occurred that increased the virulence or transmissibility of *Bd* from its ancestral state. It is possible that the *Bd* ancestor may have even been saprobic or on a different host than amphibians. The ability to grow as a cutaneous pathogen could have been the adaptation that spawned the chytridiomycosis epidemic. However, *Bd* has a number of specialized adaptations that enable it to grow intracellularly within the dynamic tissue of the epidermis, suggesting a long cutaneous history [68]. These include a life cycle length that matches the maturation of the epidermal cells and a discharge tube that opens at the skin surface. Cutaneous diseases may be more rapidly spread than sexually transmitted or blood-borne pathogens because they can be transmitted by incidental direct contact. Cutaneous fungal infections may be directly transferred from host to host without exposure to harsh environmental conditions; because of this effective means of spread and rising human population sizes, skin mycoses are globally on the rise [69]. Frog-to-frog transmission may be important because *Bd* zoospores do not have a wall and appear to lack sexual reproduction and a concomitant thick-walled resting spore [27], but see [70] for possible evidence. We hypothesize that the human mediated movement of frogs for the pet trade, scientific research, or food production followed by direct frog-to-frog transmission may have facilitated the international spread of chytridiomycosis. In addition, climate change may play a role in the transmission of the disease as, for example, longer dry seasons could have led to an increased density of frogs in ponds [71], and *Bd* is killed by high temperatures and desiccation [72],[73]. While the current pandemic appears to have occurred after the introduction of a novel pathogen into naïve host populations, the outcome of the disease is highly contingent on host as well as environmental factors.

### Conclusion

In conclusion, our data show that the current outbreak of *Bd* is due to the recent emergence of a single successful diploid lineage. We have sequenced 17 loci from 59 strains isolated from across the globe and have found no new alleles beyond the two that had already been detected in a study focused on the Sierra Nevada in California [10]. Thus these data indicate a recent and severe genetic bottleneck, apparently down to a single diploid ancestral strain. Both the extremely low genetic variation and the widespread dispersal of closely related genotypes provide strong support for the novel pathogen over the endemic pathogen hypothesis. None of the sampled regions included in this study provided strong evidence for a source population. However, increased heterozygosity of strains from North American bullfrogs may suggest that this invasive species vectored *Bd* from more diverse and healthy amphibian populations in eastern North America to ones where declines from chytridiomycosis have occurred [74],[75].

Despite the apparent absence of meiosis, the fungus harbors extensive genotypic diversity, which we hypothesize is largely caused by LOH during mitosis. No more than the global dispersal of a single diploid strain followed by LOH is needed to explain all of the diversity of existing strains. The occurrence of the same lineage in both declining populations and species and apparently stable populations supports previous results suggesting that context dependent effects such as innate host immunity and environmental factors, such as season and temperature, are critical for understanding the outcome of disease for a particular species and region [72],[76]. Following the introduction of this lineage of *Bd* into amphibian populations, some host populations have collapsed, others have crashed and then recovered, and many carry the infection with no obvious population-level effects [9],[13],[77]. The low pathogen genetic diversity and absence of frequent outcrossing imply that *Bd* may have reduced potential to coevolve with an adapting host population and could suffer from mutational meltdown [78]. Further sampling of polymorphic loci, geographic regions, and hosts will be required to test whether the current global sample is representative of true genetic diversity of the pathogen or is only the first drop of a much larger unsampled pool.

## Materials and Methods

### Ethics statement

All experiments involving animals were performed following protocols approved by the University of Maine Institutional *Animal Care and Use Committee* (Protocols A98-09-03 and A2001-09-01; JEL), Imperial College London and the British Home Office (MCF), and the Office of Laboratory Animal Care at University of Berkeley (JATM & JWT).

### Strains and collection strategy

Molecular analysis of *Batrachochytrium dendrobatidis* (*Bd*) was performed on DNA extracted from axenic cultures. Cultures were obtained from living and dead amphibians by isolation onto 1% tryptone agar using routine techniques [15]. The global sample of strains encapsulated all of the known geographic and host diversity of *Bd* represented by living cultures at the time the study was conducted. Isolation of *Bd* requires both sterile conditions and technical skill; because of this, few cultures exist in comparison with the known distribution of the disease based on histology and molecular analysis (http://www.parcplace.org/bdmap2008update.html). The sampling strategy used was therefore opportunistic and is biased towards North America for practical reasons. Most of the strains from the Morehouse et al. [27] study were included as were a subsample encompassing the genotypic diversity of the California strains from the Morgan et al. [10] study. Strains that were isolated from animals housed or shipped together were specifically eliminated in order to avoid non-independent isolates of the same clonal genotype.

### Molecular techniques

DNA was extracted from cultures grown on 1% tryptone medium with 1% agar. DNA procedures and genotyping used the protocols previously published with the following marker loci: APRT13, CTSYN1, R6046, 6164Y2, 9893X2, mb-13-8b, b7-10c, 6677X2, 6873X2, 8009X2, 8329X2, 8392X2, 8702X2 [10],[27]. An additional three loci (BdC5, BdC18, BdC24) were amplified with the following primer pairs: BdC5-F (5′-TAATAGCGCCGACCGAACTA-3′), BdC5-R (5′-ATGCCAAACCATGAGCAAAT-3′), BdC18-F (5′-GCGAATACGACTGCAAATGA-3′) BdC18-R (5′-TGAGCTCTAGCCGACATTGA-3′), BdC24-F (5′-GACAATGTGCTCACGGCTTA-3′), BdC24-R (5′-CTCTCCAAGGCTGAATCTGG-3′). Only polymorphic loci were included in this study. Complete details on the coding of sequences into diploid genotypes and the data set used to conduct genetic analyses are given in Dataset S1. This file displays the coding scheme for each of the sequence types.

An 11,026 bp fragment of the mitochondrial genome was sequenced from 16 strains: JEL203, JEL213, JEL225, JEL230, JEL239, JEL240, JEL253, JEL254, JEL258, JEL262, JEL270, JEL289, JEL404, PM-01, PM-05, PM-07. This fragment spans the intergenic region between the mitochondrial large ribosomal RNA subunit and cytochrome c oxidase subunit 1 and was amplified using long PCR with the enzyme ExTaq (Takara). Sequencing across the fragment was accomplished by primer walking with synthesized oligonucleotides. A deletion spanning 3,904 bp of DNA with no homology to any known sequences in GenBank was observed in the following four strains: JEL225, JEL262, PM-05, and PM-07.

### Data analysis

For each locus except BdC18, all of the heterozygous genotypes could be explained by the combination of the two homozygous genotypes, making assignment to alleles trivial. Only for locus BdC18 was there any ambiguity in the phasing of diploid genotypes into haplotypes, apparently due to intralocus recombination. For the purposes of analysis, locus BdC18 was divided into two loci (BdC18.1 and BdC18.2) to reflect the two regions in this marker that did not display complete linkage disequilibrium. The six genotypes observed at the locus could be completely explained by loss of heterozygosity at one or both of two polymorphic sites (BdC18.1 and BdC18.2).

The dendrogram in Figure 1 was constructed using the neighbor-joining algorithm on a distance matrix computed in PAUP v4.0b10 [79] and by statistical parsimony using TCS 1.21 ([80], Figure S1). Genetic distances between strains were calculated by considering for each locus the two different types of homozygotes two steps apart and the heterozygote 1 step from each homozygous type (hetequal coding). The data were resampled using 1,000 bootstrap pseudo-replicates to test for branch support and tested for phylogenetic signal using the permutation tail probability test in PAUP. The minimum number of outcrossing/mutation events for each locus was mapped onto the neighbor-joining phylogeny using parsimony. Ancestral state reconstruction of genotypes was inferred using MacClade 4 [49], and the number of instances incompatible with asexual reproduction recorded. For each tree, if the inferred genotype of the ancestor (root) was not heterozygous, then one mutation occurring on an internal node was allowed and not counted as an incompatibility for these trees. Calculation of Hardy-Weinberg disequilibrium, linkage disequilibrium, and tests for population differentiation were conducted using Genepop v3.4 [81]. Basic population descriptors (*H_E_* and *H_O_*) were estimated in Genetic Data Analysis 1.0d16c [82], and permutation tests were computed using custom Perl scripts. Estimates of allele richness using rarefaction were calculated using FSTAT v2.9.3.2 [83].

## Supporting Information

Figure S1 Genealogical network depicting relatedness among multilocus genotypes of Bd. The network was estimated using the software TCS 1.21 on a distance matrix calculated using “hetequal” coding of alleles (see Materials and Methods section). Each branch represents a single mutational or recombinational step. Ancestral intermediate genotypes not observed in the data set are indicated with open circles, and dashed lines indicate stretched connecting mutations.
(0.04 MB PDF)

Dataset S1 Coding scheme for the multilocus sequence typing markers and genotype matrix of global population.
(0.02 MB TXT)
