# Supplementary figures and images for: Rapid Global Expansion of the Fungal Disease Chytridiomycosis into Declining and Healthy Amphibian Populations
Source: PLoS Pathog. 2009 May 29;5(5):e1000458. doi: 10.1371/journal.ppat.1000458 (PMC2680619; doi:10.1371/journal.ppat.1000458)

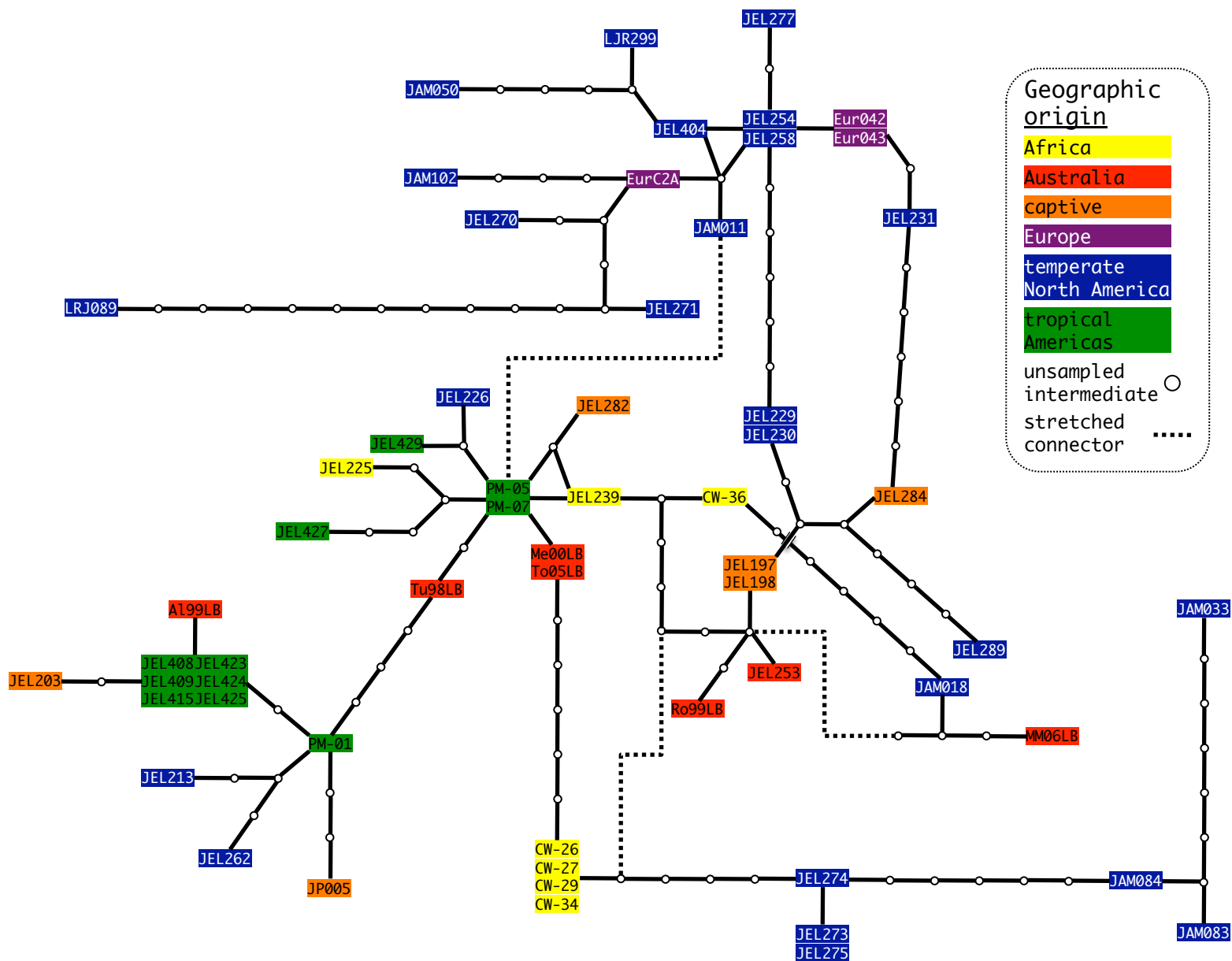

Supplement: Figure S1 — Genealogical network depicting relatedness among multilocus genotypes of Bd. The network was estimated using the software TCS 1.21 on a distance matrix calculated using “hetequal” coding of alleles (see Materials and Methods section). Each branch represents a single mutational or recombinational step. Ancestral intermediate genotypes not observed in the data set are indicated with open circles, and dashed lines indicate stretched connecting mutations. (0.04 MB PDF) [file ppat.1000458.s001.pdf]
